# Mediterranean Journal of Rheumatology September 2018 Highlights

**DOI:** 10.31138/mjr.29.3.118

**Published:** 2018-09-27

**Authors:** Dimitrios P. Bogdanos

**Affiliations:** Department of Rheumatology and Clinical Immunology, Faculty of Medicine, School of Health Sciences, University of Thessaly, Larissa, Greece

In this issue of the Journal, original papers, reviews, case studies and research protocols related to the broad spectrum of rheumatic diseases are published. All of them are of interest, discuss novel issues and significantly contribute to the ongoing translational research and clinical practice in the field of rheumatology.

In their original article, Sakkas and collaborators report the multiparametric autoantibody profile of their patients with systemic sclerosis (SSc) and morphea.^1^ What is new in this single-centre study is the appreciation that patients with morphea have entirely distinct autoantibody profile from patients with SSc. This study, which is the most comprehensive analysis of autoantibody profiling of 13 systemic sclerosis related autoantibodies in Greek patients with systemic sclerosis, points towards the need to include anti-RNA pol III antibodies in the routine testing of patients with clinical suspicion of systemic sclerosis as this increases the diagnostic sensitivity of autoantibody detection. It suggests that if anti-CEN, anti-Topo I and anti-RNA pol III antibodies are negative but the clinical suspicion remains high, physicians may need to assess for the remaining autoantibody specificities by specialized laboratories.

Ziaragkali et al.^2^ bring to our attention a common problem that we have to face in many patients with autoimmune rheumatic diseases: Dry eye is extremely frequent in our patients, irrespectively of whether they have Sjögren’s Syndrome or not. The Authors discuss issues related to the approach that we can/must have in patients presenting with this problem in routine clinical practice.

Ahmed et al.^3^ comprehensively reviewed the literature searching the Medline database using as keywords ‘systemic sclerosis’ and ‘interstitial lung disease, ILD’. Their aim was to discuss ILD in SSc in depth, touching upon the recent advances on pathogenesis, biomarkers, prognosticators and outcomes but most importantly to update us where we stand in terms of efficient treatment approaches. The authors conclude that despite ongoing research, an urgent need for novel approaches is required, as fibrosis appears irreversible.

In their review, Christoforidou and Galanopoulos provide an overview of diffuse connective tissue disorders in human immunodeficiency virus (HIV)-infected individuals.^4^ The Authors underline the need for increased awareness in interpreting clinical and laboratory findings in these patients. Clinicians dealing with these patients must be able to discriminate actual rheumatological disorders from non-specific manifestations of acute and chronic HIV infection.

The implementation of the transition process in patients with autoimmune rheumatic diseases is not an easy task. Pratsidou and collaborators review this emerging topic and describe the coordinated, cooperative plan required by the transition team, the patient and its family.^5^ The Authors take us through the general frames for transition issued by the American College of Physicians, the American College of Rheumatologists and the EULAR/Paediatric Rheumatology European Society (PReS) Transition recommendations. More importantly, however, the authors, stemming from their long-lasting experience, discuss the implementation of these policies taking into consideration the local infrastructure and the family/patient cultural background and the fact that caregivers of Mediterranean countries differ from Northern European countries. Their recommendations underscore the mutual special handling of the transition, and the gradual multi-task education.

In their case study, Marketos et al.^7^ describe a young woman with rheumatoid arthritis with fever and rapid, destructive joint involvement, who responded to Canakinumab administration after treatment failure to a series of medications including methotrexate and leflunomide, anti-TNF, IL-6 inhibitor, B cell depletion and IL-1RA. The Authors discuss the need for rheumatologists to be alert of an IL-1β-driven disease especially at early disease courses. In another case report, Panagopoulos and Katsifis, describe a case of hemophagocytic lymphohistiocytosis (HLH), which developed in a 58-year-old woman with seropositive rheumatoid arthritis (RA), who presented with fever and signs of vasculitis.^6^ Treatment with corticosteroids and intravenous immunoglobulin led to resolution of fever, management of pancytopenia and complete healing of the ulcers. The case helps the Readers of the Journal, especially the less experienced ones, to familiarize themselves with HLH and to understand that accurate diagnosis and proper intervention may lead to favorable outcome. Argyropoulou et al.^8^ in their research protocol will extensively assess the phenotype of vascular damage in non-infectious primary vasculitides (NIPV) with the use of non-invasive vascular biomarkers, a topic of great interest. Daoussis and collaborators, in the outline of their research porotocol supported by ERE-EPERE, point the need to assess the clinical efficacy of adrenocorticotropic hormone (ACTH) in hospitalised patients and to compare it to betamethasone.^9^ In their randomized controlled trial they will also investigate the safety profile of ACTH vs betamethasone and the effect of ACTH on immune responses and metabolic parameters. According to the Authors, if the efficacy and safety profile of ACTH is proven, the use of ACTH will be strongly supported for the treatment of gout in hospitalized patients with significant comorbidities receiving multiple medications.

We hope that the papers of this issue will be of interest to the audience of the Journal and we are committed to reiterate our focus on inspiring papers in our next issue. Enjoy the reading.
